# Welcome to *PLoS Computational Biology* “Education”

**DOI:** 10.1371/journal.pcbi.0020007

**Published:** 2006-01-27

**Authors:** Fran Lewitter

The past decade has witnessed a dramatic increase in the availability of both biological information and tools for scientists. These tools and the methods by which they are applied are commonly used in all phases of biological research, for example, using BLAST (basic local alignment search tool) to find putative homology between large varieties of sequences from different species or using a metaserver combining several methods to perform secondary and tertiary protein structure prediction from primary sequences.

Not surprisingly, the computational literacy of biologists has increased, with many lab biologists now willing and able to write and function in a new, specifically UNIX, environment. As they start to work within this environment, they begin to share more and more common ground with the active computational biology community. No matter what level of expertise one seeks in bioinformatics and computational biology, it is important that all individuals on both the biology and the computational side share a common vocabulary. In doing so, we may better supervise and communicate with others who use these tools.

Recognizing the need for training and education in bioinformatics and computational biology specifically targeted to biologists, *PLoS Computational Biology* launches “Education,” a new column with several goals. The first goal is to provide both practical and background information on important computational methods used to investigate interesting biological questions. The second goal is to provide information in the column that may be used at your institution for training others. Finally, we hope to stimulate widespread interest in an expanding field. To begin to meet these goals, we will focus initially on providing both historical reviews and tutorials.

An important part of education is an appreciation of the historical perspective of the field. To provide this history, we envision articles that look at the evolution of database search techniques and of techniques and algorithms for multiple sequence alignment, as well as at reviews of open-source structural prediction software, computational methods for predicting transmembrane proteins, and computational methods for comparative genomics. As an example, later in the year, Kimmen Sjolander, from the University of California Berkeley, will be contributing a review on phylogenetic reconstruction of protein superfamilies.

The tutorials we present will concentrate on biological questions, followed by a discussion that covers the relevant computational tools to help answer those questions. We want to address topics such as how to set up a genome browser to use with your data, what transcription factors regulate your genes of interest, how to set up and query a relational database of biological information, and what parts of your favorite protein are most conserved.

Our first tutorial, “Practical Strategies of Motif Discovery,” will be contributed by Ernest Fraenkel and Kenzie MacIsaac from the Massachusetts Institute of Technology. Later in the year, we hope to publish some of the tutorials presented at the annual Intelligent Systems for Molecular Biology Meeting organized by the International Society for Computational Biology (ISCB). The 2006 annual meeting will be held in Fortaleza, Brazil, in early August (http://ismb2006.cbi.cnptia.embrapa.br).

Tutorials and reviews are only the beginning. Over time, we will explore ways to present educational information in this digital age that can take advantage of technological innovation. In addition to text-based information, we are considering multimedia presentations and other media to enhance the written word. On this front, in particular, we welcome any comments and suggestions from the community.

As Education Editor, my approach to the column draws from my experience working with many scientists at Whitehead Institute for Biomedical Research. For more than a decade, I have been providing bioinformatics education, support, consultation, and collaboration for this community. My membership with the ISCB Education Committee also provides a source of material for the column. I encourage participation by this active group, with participants from around the world, in developing and contributing to this column. But most importantly, I invite all our readers who have ideas for a review or tutorial and who would like to write it to please send them our way.

